# Visualization of ligand-induced dopamine D_2S_ and D_2L_ receptor internalization by TIRF microscopy

**DOI:** 10.1038/s41598-017-11436-1

**Published:** 2017-09-07

**Authors:** Alina Tabor, Dorothee Möller, Harald Hübner, Johannes Kornhuber, Peter Gmeiner

**Affiliations:** 1 Department of Chemistry and Pharmacy, Emil Fischer Center, Friedrich-Alexander University Erlangen-Nürnberg, Schuhstraße 19, 91052 Erlangen, Germany; 2 Department of Psychiatry and Psychotherapy, Friedrich-Alexander University Erlangen-Nürnberg, Schwabachanlage 6, 91054 Erlangen, Germany

## Abstract

G protein-coupled receptors (GPCRs), including the dopamine receptors, represent a group of important pharmacological targets. Upon agonist binding, GPCRs frequently undergo internalization, a process that is known to attenuate functional responses upon prolonged exposure to agonists. In this study, internalization was visualized by means of total internal reflection fluorescence (TIRF) microscopy at a level of discrete single events near the plasma membrane with high spatial resolution. A novel method has been developed to determine the relative extent of internalized fluorescent receptor-ligand complexes by comparative fluorescence quantification in living CHO cells. The procedure entails treatment with the reducing agent sodium borohydride, which converts cyanine-based fluorescent ligands on the membrane surface to a long-lived reduced form. Because the highly polar reducing agent is not able to pass the cell membrane, the fluorescent receptor-ligand complexes located in internalized compartments remain fluorescent under TIRF illumination. We applied the method to investigate differences of the short (D_2S_) and the long (D_2L_) isoforms of dopamine D_2_ receptors in their ability to undergo agonist-induced internalization.

## Introduction

G protein-coupled receptors (GPCRs) represent a large family of integral membrane proteins and their primary function is to transduce extracellular stimuli (e.g ligand binding) into intracellular signals^1^. These receptors play fundamental roles in many physiological and pharmacological processes and therefore serve as important drug targets which are addressed by more than 30% of the current drugs on the market^2^. Dopamine D_2_ receptors, which belong to the family of class A GPCRs, mediate various physiological functions and are known as valuable targets for the treatment of neuropsychiatric disorders including schizophrenia, Parkinson’s disease and drug addiction^3–8^.

D_2_ receptors exist in two splice variants, the short (D_2S_) and the long (D_2L_) isoform, which have been suggested to be localized pre- and postsynaptically, respectively^9^. Although different spatial distributions and functions have been suggested, the individual contributions of the two isoforms to (patho)-physiological processes remain unclear.

Following agonist stimulation, GPCRs undergo conformational changes that allow binding and activation of heterotrimeric G proteins. Subsequently, the Gα and Gβγ subunits modulate a variety of signaling events within the cell. Importantly, GPCRs undergo a highly dynamic regulation upon activation. Multiple mechanisms provide control over the specificity and extent of the cellular response. For instance, agonist stimulation of receptors at the cell surface can induce receptor desensitization and, consequently, receptor internalization through different endocytotic pathways^10, 11^. Agonist activation of most GPCRs leads to phosphorylation of the receptors by G protein-coupled receptor kinases (GRKs). GKRs and β-arrestins, together orchestrate receptor uncoupling from the G protein and thereby terminate G protein signaling^12, 13^. In many cases, β-arrestin recruitment leads to receptor internalization, which generally proceeds by clathrin-coated pits or other mechanisms of endocytosis^14–16^, in which the receptors are translocated away from the plasma membrane to lager intracellular vesicular or endosomal structures. Once internalized, GPCRs are either dephosphorylated and recycled back to the plasma membrane, or targeted to lysosomes for proteolysis, which results, in part, in receptor downregulation^17^. Interestingly, recent reports have demonstrated that canonical GPCR signaling may also occur from internalized GPCRs in endosomes^18–21^.

Various experimental approaches have been utilized to measure GPCR ligand binding and internalization. Among them are, in particular, radioligand binding studies, enzyme-linked immunoabsorbent assays (ELISA) and fluorescent immunocytochemistry experiments^22–24^. However, as a principal limitation these protocols generally require fixation and/or permeabilization of cells. Other approaches for directly measuring receptor internalization include microplate-based functional cell assays build upon enzyme fragment complementation, bioluminescence- and fluorescence-resonance energy transfer technologies (BRET and FRET)^25–27^. Moreover, alternative strategies based on fluorescence microscopy using fluorescent protein tags have been followed to monitor internalization in living cells^28, 29^.

Fluorescence microscopy facilitates a novel way to study ligand binding and the subsequent internalization process of GPCRs in living cells, using fluorescence-based probes such as fluorescent ligands^30^. In particular, total internal reflection fluorescence (TIRF) microscopy has proved useful to study internalization because of its ability to selectively detect both fluorescent molecules situated within or in close proximity (~100 nm) to the plasma membrane with high spatial and temporal resolution^18, 31, 32^.

Taking advantage of our recently developed high affinity fluorescent dopamine receptor agonists and antagonists^33^, we visualize internalization by means of TIRF microscopy at a level of discrete single events near the plasma membrane. Employing the reducing agent sodium borohydride (NaBH_4_), which converts cyanine-based fluorescent dyes on the membrane surface to a long-lived reduced form, we herein present a novel method to determine the relative proportion of internalized fluorescent receptor-ligand complexes by comparative fluorescence quantification under living cell conditions. Using this method, we are able to investigate differences of the two dopamine D_2_ receptor isoforms, D_2S_ and D_2L_, in their ability to undergo agonist-induced internalization.

## Results

### Direct visualization of agonist-induced cluster formation of D_2S_ and D_2L_ receptors

**Figure 1:**
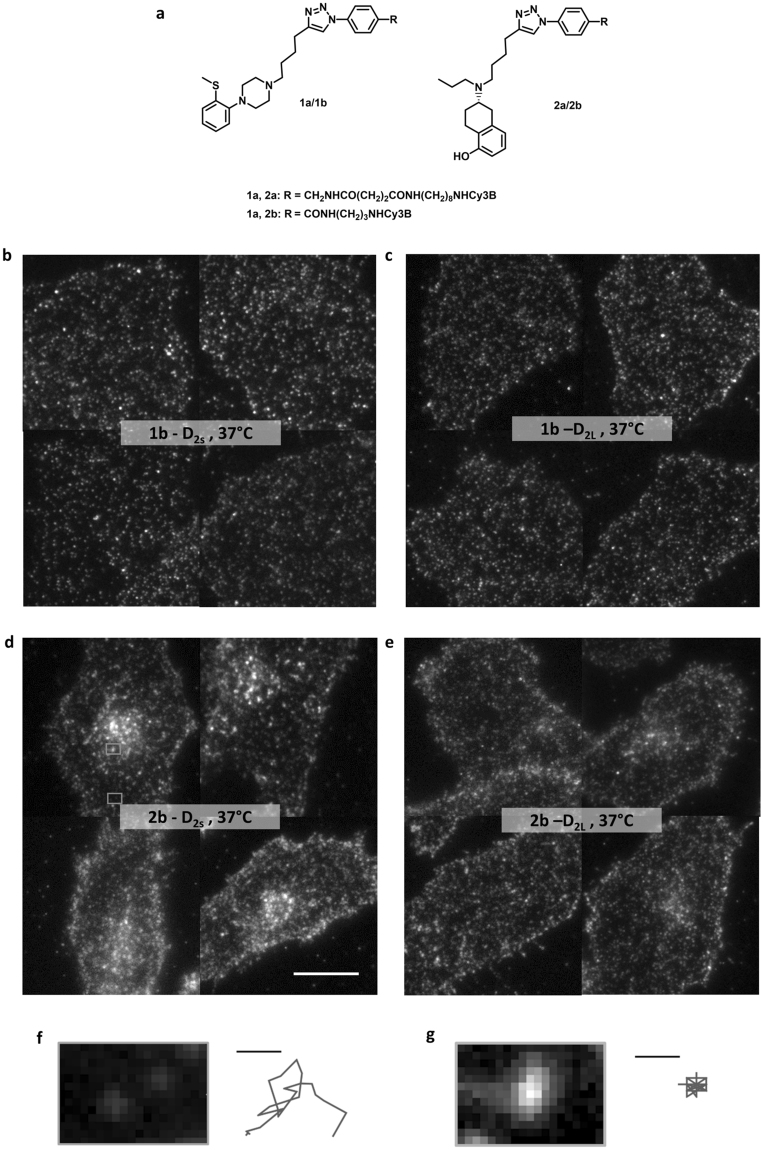
Visualization of agonist-induced dopamine D_2S_ and D_2L_ receptor fluorescent cluster formation in stably transfected CHO cells. (**a**) Chemical structure of the fluorescent antagonists (1a,b) and agonists (2a,b). (**b–e**) Representative TIRF images of CHO cells stably expressing D_2S_ or D_2L_ receptors exposed to the fluorescent antagonist 1b (10 nM) or agonist 2b (10 nM) for 1 h at 37 °C. Scale bar, 10 µm. (**f,g**) Intensity and position (left) and tracked path (right) of the selected fluorescent spots revealed different diffusive behavior (**f**, single ligand-receptor complexes in the cell membrane; **g**, cluster of ligand receptor complexes). Scale bar, 1 µm.

Very recently, we reported on the pharmacological characterization of Cy3B-conjugated fluorescent antagonists (**1a**,**b**) and agonists (**2a**,**b**) targeting dopamine D_2_/D_3_ receptor subtypes (Fig. 1a)^33^. These four fluorescent ligands were shown to possess binding affinities in the low nanomolar range (Supplementary Table S1) and their suitability for live-cell fluorescence microscopy with excellent signal to noise ratios and high temporal and spatial resolution could be demonstrated^33^.

Initial TIRF microscopy experiments in this study showed that receptor-ligand complexes of the fluorescent antagonists **1a** and **1b** bound to dopamine D_2S_ or D_2L_ receptors stably expressed in CHO cells (10 nM, for 1 h at 37 °C) were visible under TIRF illumination as individual, freely diffusing, diffraction limited spots. These spots were homogeneously distributed over the membrane of the living CHO cells (representative for **1b**, see Fig. 1b,c, Supplementary Movie S1). Interestingly, cells incubated with the fluorescent agonists **2a**,**b** showed an inhomogeneous labeling distribution (representative for **2b**, see Fig. 1d,e, Supplementary Movie S2). In addition to the labeled receptors on the membrane surface, fluorescent puncta were observed, which were predominantly clustered. In contrast to the diffraction-limited spots of single ligand-receptor complexes (Fig. 1f), these fluorescent puncta could not be treated as diffraction-limited spots and showed confined diffusive behavior and increased intensities (Fig. 1g). Moreover, the TIRF experiments revealed that the short and long isoforms of the D_2_ receptors (D_2S_ and D_2L_) differ in their ability to form the clustered fluorescent puncta (Fig. 1d,e). These findings could not be attributed to differences in receptor expression as the experiments were performed with monoclonal CHO cell lines with nearly identical expression levels of the D_2S_ or D_2L_ receptors, respectively (B_max_ of 970 ± 60 fmol mg^-1^ protein for D_2S_ and 1060 ± 60 fmol mg^−1^ protein for D_2L_)^33^.

To further investigate this initial observation, CHO cells stably expressing D_2S_ receptors were pretreated with an excess of the unlabeled antagonist spiperone (10 µM, 2 h at 37 °C), followed by the incubation with the fluorescent agonist **2b** (10 nM, 1 h at 37 °C). When these cells were imaged under TIRF illumination, neither clustered fluorescent puncta nor labeling of the receptors at the cell surface were observed, indicating that unspecific binding and uptake of ligand **2b** were negligible (Supplementary Fig. S1). Thus, we hypothesized that the fluorescent agonists **2a**,**b** were internalized into the living CHO cells in a dopamine D_2_ receptor specific manner.

### ELISA-detected internalization and β-arrestin recruitment

**Figure 2:**
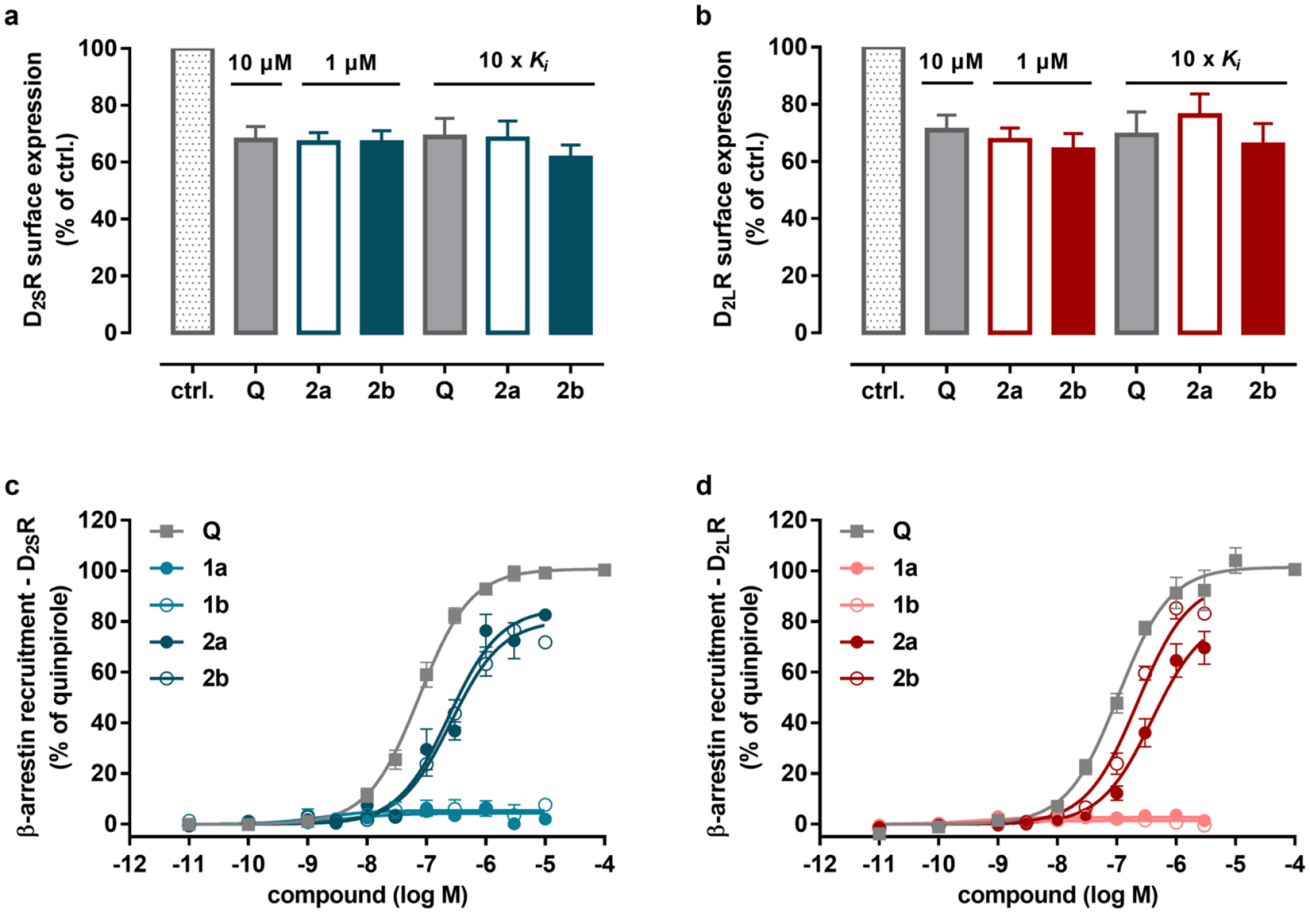
Agonist-induced internalization by cell surface ELISA and β-arrestin-2 recruitment at D_2S_ and D_2L_ receptors. N-terminally FLAG-tagged D_2S_ (**a**) and D_2L_ (**b**) receptors were transiently expressed in HEK cells and surface expression was examined after treatment with the reference agonist quinpirole (Q) or the fluorescent agonists (**2a**,**b**) for 1 h at 37 °C with the concentrations indicated. All ligands induced significant receptor internalization compared to vehicle (ctrl., one way-ANOVA followed by Dunnet’s posthoc test, p < 0.05). Data represent mean ± s.e.m. of n ≥ 4 independent experiments, each performed in quintuplicates. β-Arrestin-2 recruitment was determined employing the DiscoverX PathHunter assay. HEK cells stably expressing β-arrestin-2 tagged with the EA-fragment were transfected with ProLink-tagged D_2S_ (**c**) or D_2L_ (**d**) receptors, respectively. The fluorescent agonists **2a** and **2b** stimulate β-arrestin-2 recruitment dose-dependently and display almost full agonist activity. The fluorescent antagonists **1a** and **1b** do not induce β-arrestin-2 recruitment. Data represent mean ± s.e.m of n ≥ 3 independent experiments, each performed in duplicate. Results were normalized to the maximum effect of the reference agonist quinpirole (100% for D_2S_ and D_2L_ receptors).

In order to associate our initial findings, the significant agonist-induced relocalization of D_2_ receptors, with the GPCR internalization process, we used a classical ELISA (enzyme-linked immunosorbent assay) approach^34^. Therefore, the decrease of cell surface expression of FLAG-tagged D_2S_ or D_2L_ receptors was quantified in response to treatment with fluorescent agonists (**2a**,**b**) in transiently transfected HEK cells (Fig. 2a,b). At a concentration of 1 µM, both fluorescent agonists induced internalization of dopamine D_2S_ and D_2L_ receptors, respectively, highly similar to a reference agonist quinpirole (10 µM) (remaining surface expression for D_2S_: 68 ± 5% quinpirole, 67 ± 3% **2a**, 67 ± 4% **2b**; and D_2L_: 71 ± 5% quinpirole, 67 ± 4% **2a**, 64 ± 6% **2b** (mean ± s.e.m)). Comparable results were also obtained when concentrations identical with those used within TIRF microscopy experiments (10-fold of *K*_i_ concentration) were applied in the internalization assay (remaining surface expression for D_2S_: 69 ± 7% quinpirole, 68 ± 3% **2a**, 62 ± 5% **2b**; and D_2L_: 69 ± 8% quinpirole, 76 ± 8% **2a**, 66 ± 7% **2b** (mean ± s.e.m), Fig. 2a,b).

Although different mechanisms ultimately leading to receptor internalization have been described, we were interested if our fluorescent agonists are able to engage the most prevalent and best characterized pathway for GPCR desensitization: the recruitment of β-arrestins to ligand-activated receptors^35–37^. Employing a commercially available test system based on enzyme-fragment complementation (DiscoverX PathHunter assay), we found that the agonists **2a** and **2b** stimulate substantial recruitment of β-arrestin-2 at D_2S_ and D_2L_ receptors(D_2S_: EC_50_ = 240 ± 70 nM, E_max_ = 87 ± 4% and EC_50_ = 230 ± 60 nM, E_max_ = 75 ± 7%, for **2a** and **2b**, D_2L_: EC_50_ = 400 ± 60 nM, E_max_ = 83 ± 7%, and EC_50_ = 230 ± 30 nM, E_max_ = 96 ± 4%, for **2a** and **2b** (mean ± s.e.m)), while the antagonists **1a** and **1b** were devoid of intrinsic activity (Fig. 2c,d and Supplementary Table S1). Compared to the reference agonist quinpirole (D_2S_: EC_50_ = 79 ± 9 nM, E_max_ = 100 ± 1% and D_2L_: EC_50_ = 110 ± 10 nM, E_max_ = 100 ± 1% (mean ± s.e.m)) both, efficacies and potencies were found to be slightly reduced. Together, the results from ELISA-based internalization assays and β-arrestin-recruitment studies indicate that the fluorescent agonists **2a**,**b** are able to stimulate receptor desensitization and trafficking while the fluorescent antagonists **1a**,**b** do not induce these processes. Thus, we hypothesized that the occurrence of fluoresccent puncta observed by TIRF microscopy in presence of the agonists **2a**,**b** but not the antagonists **1a**,**b** may be related to these processes.

### Control of clustered receptor-ligand complexes by temperature

Several studies have shown that endocytosis and trafficking mechanisms are blocked at reduced temperatures^38, 39^. Indeed when we decreased the incubation temperature from 37 °C to ambient temperature (22–24 °C), the number of intracellular fluorescent puncta was significantly reduced after treatment of D_2S_ and D_2L_ with the fluorescent agonist **2b** (10 nM, Supplementary Fig. S2). The spot density corresponding to **2b**-D_2L_ complexes at the cell surface (0.65 ± 0.03 spots µm^−2^, mean ± s.d. of 10 cells) was found to be highly comparable to that of antagonist **1b**-D_2L_ complexes (0.67 ± 0.03 spots µm^−2^, mean ± s.d. of 10 cells, Fig. 1c).

### Determination of the relative extent of internalized receptor-ligand complexes

**Figure 3:**
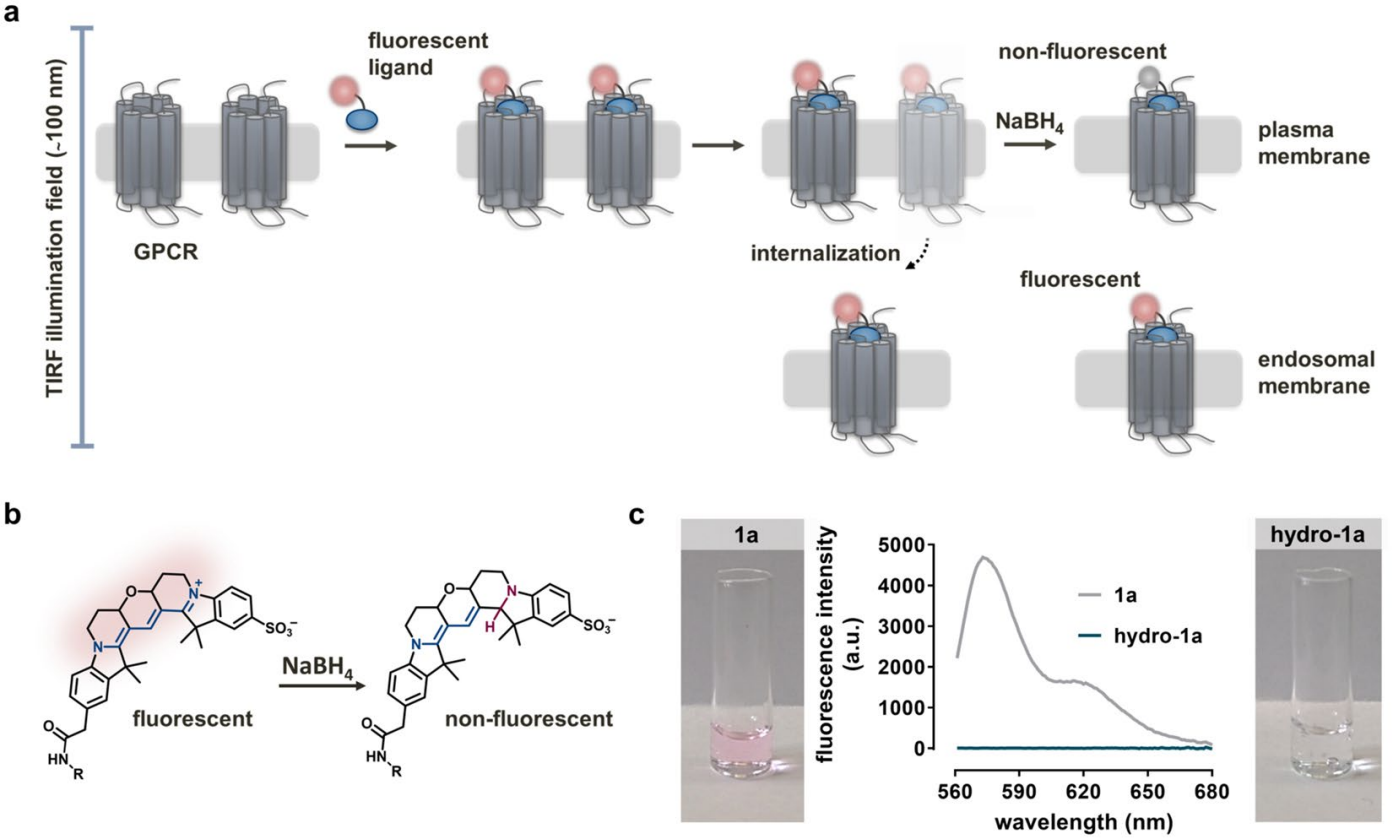
Reductive treatment approach. (**a**) Schematic illustration of the reductive treatment approach for the determination of the relative proportion of internalized receptor-ligand complexes. (**b**) Proposed structure of the non-fluorescent hydrocyanine after treatment of the fluorescent cyanine (Cy3B) with NaBH_4_. (**c**) Spectroscopic validation of the reductive treatment of the Cy3B-conjugated fluorescent ligand **1a** with NaBH_4_. Emission spectra (λ_ex_ = 530 nm) of the Cy3B-conjugated ligand **1a** in the presence and absence of NaBH_4_ (30 mM) in dPBS. **Hydro-1a** shows negligible fluorescence emission.

To further explore the origin of the differences in the ability of D_2S_ and D_2L_ receptors to form clustered fluorescent puncta upon fluorescent agonist-binding, we developed a new method allowing to distinguish receptors on the cell surface from receptors internalized to intracellular compartments in living cells. The procedure entails treatment with the reducing agent sodium borohydride (NaBH_4_), which converts the cyanine-based fluorescent dyes to a long-lived reduced form. Because the highly polar NaBH_4_ is not able to pass the cell membrane, ligands bound to internalized receptors should be not affected by the treatment. Thus, the internalized Cy3B-conjugated ligands located in internalized compartments (endosomes) should remain fluorescent under TIRF illumination (Fig. 3a).

In order to prove that the fluorescence intensity of the Cy3B-conjugated ligands is negligible after reduction with NaBH_4_, we performed *in vitro* control experiments. Thus, we added NaBH_4_ (final concentration 30 mM) to an aqueous solution of the fluorescent antagonist **1a** (1 µM in dPBS) and measured an emission scan (λ_ex_ = 530 nm) with a spectrofluorimeter after 5 min. In agreement with previous reports on the reduction of cyanine dyes^40, 41^, the hydro-form of **1a** displayed non-fluorescent properties (Fig. 3b,c).

**Figure 4:**
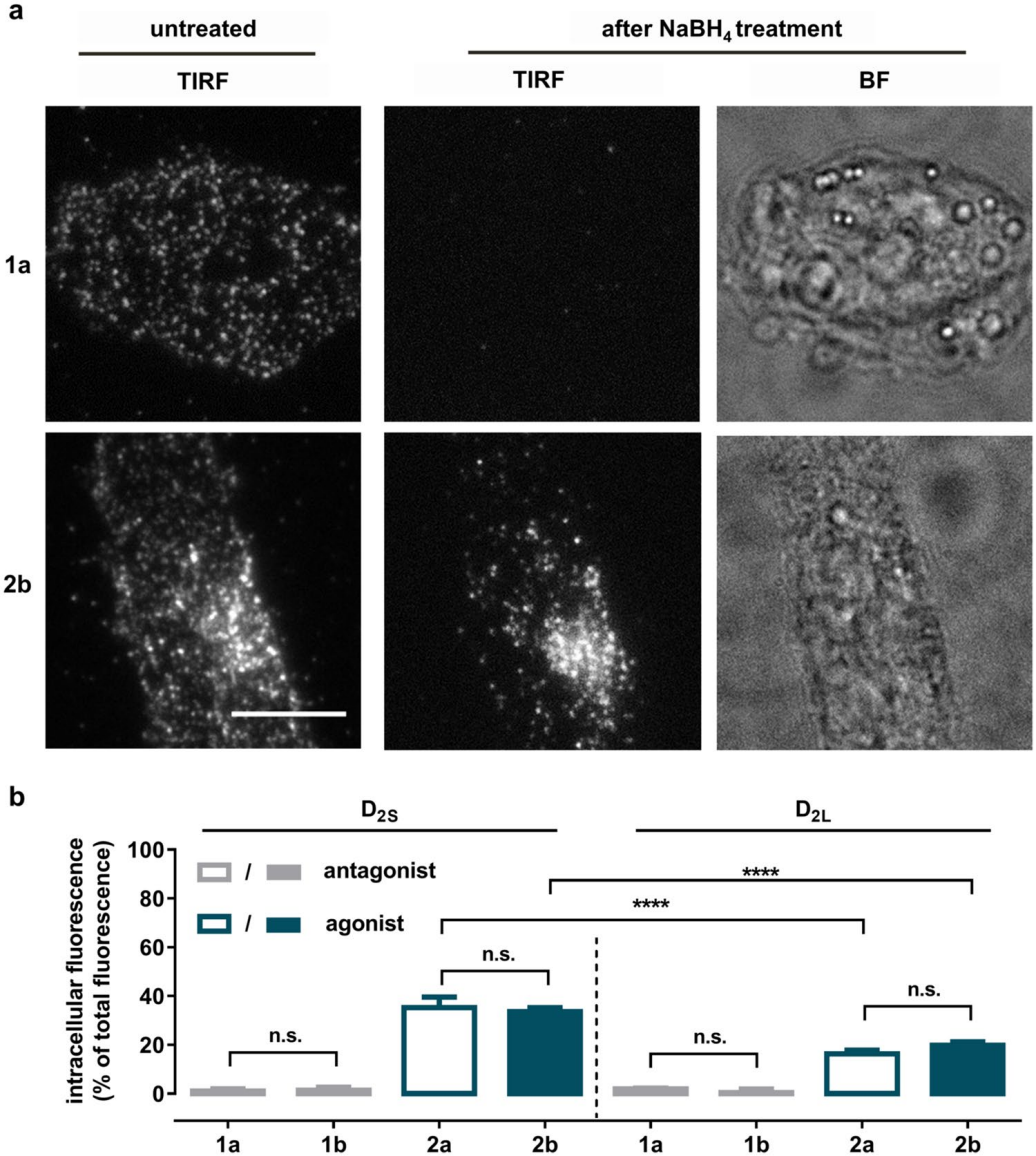
Ligand-induced internalization of D_2L_ and D_2S_ receptors. (**a**) Validation of the reductive treatment approach of **1a** and **2b** labeled D_2S_ receptors in living cells by TIRF microscopy. TIRF and brightfield (BF) images of CHO cells stably expressing the D_2S_ receptor incubated with the fluorescent ligand **1a** and **2b** for 1 h at 37 °C before and after treatment with NaBH_4_. Scale bar, 10 µm. (**b**) Determination of the relative extent of internalized fluorescent receptor-ligand complexes. CHO cells stably expressing D_2S_ or D_2L_ receptors, respectively, were incubated for 1 h at 37 °C with the corresponding fluorescent ligands (concentration corresponding to the tenfold of the respective *K*_*i*_ value) and were imaged under TIRF illumination before and after NaBH_4_ treatment (30 mM, 5 min). Data represent the fraction (%, mean ± s.e.m) of NaBH_4_ inaccessible (internalized) mean fluorescence intensity of a fluorescently-labeled cell relative to the total cell’s mean fluorescence intensity before the NaBH_4_ treatment. At least three independent experiments were performed, with four or more cells imaged per condition. Statistical analysis was performed by an unpaired *t*-test, **** *p*-value < 0.0001, n.s. - not significant).

To demonstrate the suitability of the reductive treatment procedure under living cell conditions, CHO cells stably expressing the D_2S_ receptor were incubated with the fluorescent ligands **1a** (antagonist) and **2b** (agonist) at a concentration of 10 nM for 1 h, followed by the treatment with 30 mM NaBH_4_ for 5 min. Images of the cells were acquired before and after NaBH_4_-treatment under TIRF illumination (Fig. 4a). As expected, for the fluorescent antagonist **1a** treatment with NaBH_4_ reduced the initial fluorescence corresponding to receptor surface-bound fluorescent ligands to a nearly undetectable level. However, TIRF images of **2b**-labeled cells after NaBH_4_ treatment revealed fluorescent puncta, indicating that the fluorescence results from reduction-resistant **2b**-receptor complexes localized in intracellular compartments (Fig. 4a).

The dynamics of these internalized compartments under TIRF illumination suggests that the cell treatment with NaBH_4_ has minor effects on the cell viability (Supplementary Movie S3). Bright field images of the NaBH_4_-treated cells also showed no significant changes in cell morphology or adhesive behavior.

To determine the relative extent of internalization, the reductive treatment approach was applied and evaluated by fluorescence quantification. Therefore, CHO cells stably expressing either D_2S_ or D_2L_ receptor were incubated for 1 h with the corresponding fluorescent ligands (**1a**,**b** or **2a**,**b**) at a concentration corresponding to the tenfold of the *K*_*i*_ value at 37 °C (Supplementary Table S1). Cells of each condition were imaged under TIRF illumination before and after NaBH_4_ treatment.

The mean fluorescence intensity of a fluorescent ligand-labeled cell after NaBH_4_ treatment was used to quantify fluorescence in the intracellular TIRF illumination field. The ratio of the intracellular fluorescence and the total fluorescence of the same cell before NaBH_4_ treatment was used as a measure of receptor internalization. Importantly, TIRF microscopy only visualizes the peripheral cytoplasm (~100 nm from the basolateral membrane) and thus cannot be used to quantify the total amount of internalized receptors within an entire cell. However, the relative ratio of intracellular to total fluorescence can be applied to compare the internalization behavior of two different systems (e.g. receptor subtypes or ligands).

Figure 4b summarizes the results of the experiments described above. The extent of agonist-induced D_2S_-mediated internalization was found to be significantly higher (**2a**: 35.3 ± 4.2% and **2b**: 32.9 ± 1.9% (mean ± s.e.m.)) than for the D_2L_ receptor isoform (**2a**: 16.3 ± 1.5% and **2b**: 19.8 ± 1.5% (mean ± s.e.m.)). The fluorescent antagonists **1a** and **1b** were not able to promote D_2S_ and D_2L_ receptor internalization (D_2S_-**1a** 1.1 ± 0.9%, D_2S_-**1b** 1.4 ± 1.3%, D_2L_-**1a** 1.9 ± 0.4% and D_2L_-**1b** 0.4 ± 1.5% (mean ± s.e.m)).

## Discussion

There are still many open questions regarding the regulatory mechanisms involved in the downregulation of activated GPCRs including receptor internalization and signal termination^18–21^. Some of these questions can be addressed by fluorescence single-molecule imaging of GPCRs and their signal transducers with suitable fluorescent probes^18, 42^. In particular, TIRF microscopy offers the possibility of studying membrane proteins with higher spatial and temporal resolution than conventional epifluorescence or confocal fluorescence microscopy. Moreover, events occurring within the plasma membrane like receptor-dimerization^33, 43, 44^ or in close proximity to the membrane such as internalization can be studied in living cells under nearly physiological conditions^32, 45^.

Very recently, we have demonstrated that the fluorescent dopamine receptor antagonists **1a**,**b** and agonists **2a,b** can be used to directly visualize ligand binding to GPCR monomers and dimers with single molecule resolution by TIRF microscopy^33^. In the present study, we found that D_2_ expressing CHO cells labeled with the antagonists **1a**,**b** showed a homogenous spot density and distribution, while our experiments with the agonists **2a**,**b** revealed the formation of clustered fluorescent puncta under TIRF illumination. Using a new approach based on the treatment with the mild reducing agent NaBH_4_, we were able to convert fluorescent ligands bound to receptors at the cell surface to a non-fluorescent dark state^40, 41^. In contrast, the clustered fluorescent puncta were inaccessible for the polar NaBH_4_ and remained fluorescent. Since the fluorescent agonists **2a**,**b** stimulate substantial β-arrestin-2 recruitment and a comparable degree of receptor internalization in HEK cells transiently transfected with D_2S_ or D_2L_ receptors, we attributed these fluorescent clusters to internalized receptor ligand complexes.

Up to date, several methods have been employed to study receptor internalization. Very frequently, radioligand binding studies, ELISA- or immunofluorescence-based techniques and microplate-based functional whole cell assays have been used to quantify the proportion of internalized receptors^22–29^. Each of these methods has its own advantages and limitations. Classical radioligand binding studies require the availability of radioligands with distinct polarity and thus membrane permeability^24, 46^ to determine the fraction of extra- and intracellular receptors. In contrast, the surface-bound fraction of a radioligand or an antibody is removed under acidic conditions in the “acid wash” method. The remaining cell-bound fraction is considered to be internalized in acid-resistant compartments such as endosomes. Although this method has been widely used to investigate ligand-dependent GPCR internalization^47–50^, it is not particularly suitable for dynamic analyses and fluorescence quantification, since not all compounds are washed off easily, requiring harsh treatment of the cells and cell membrane permeabilization^47, 51^. Other methods such as ELISA or immunofluorescence require highly specific antibodies or adequately tagged receptors, complicating or even excluding their application to native cells or tissue. In addition, cells usually have to be fixed and/or permeabilized. Although intact living cells are used in microplate-based whole cell functional assays on first hand, the detection step often requires cell lysis. Moreover, these bulk measurements preclude single-cell or subcellular resolution.

The TIRF imaging approach in combination with our high affinity fluorescent dopamine receptor agonists and antagonists is compatible with the physiological conditions of ligand-receptor interaction, allowing the investigation of D_2_ receptor internalization in living cells. Receptors are visualized in one single labeling step allowing for the monitoring of ligand binding and receptor trafficking. Treatment with NaBH_4_ represents a mild, fast and efficient method to distinguish receptors on the cell surface from those in intracellular compartments without causing obvious changes in cell morphology. The relative extent of receptor internalization can be determined by comparison of the overall fluorescence intensity of the same living cell before and after treatment with the reducing agent. Although we have used CHO cells stably expressing our receptors of interest (D_2S_ and D_2L_), TIRF microscopy using fluorescent ligands is generally applicable to cells endogenously expressing GPCRs^52^. Limitations of the approach include the relatively low throughput and the requirement of suitable fluorescently labeled agonists. Further, it should be acknowledged that due to the low penetration depth of the evanescent field (~100 nm) only a relative and no absolute quantification of receptors can be obtained.

Taking advantage of our new methodology, we have observed that the dopamine D_2_ receptor isoforms, D_2S_ and D_2L_, stably expressed in CHO cells, show differences in their internalization behavior. While approximately 32–35% of the detected D_2S_ receptors were found to be localized in intracellular compartments, only 16–20% of the observed D_2L_ receptors were found to internalize upon incubation with the fluorescent agonists **2a**,**b** for 1 h at 37 °C. Consistent with the present results, a number of previous studies have described that D_2S_ and D_2L_ receptors behave differently in their sensitivity to internalization/desensitization processes^24, 34, 46^. For instance, Itokawa *et al*. demonstrated in radioligand binding studies with stably transfected CHO cells that about 44% of surface expressed D_2S_ receptors became unavailable for the hydrophilic radioligand [^3^H]sulpiride after incubation with the endogenous agonist dopamine. The internalization of D_2L_ was not only lower (22%), but also proceeded significantly slower (half-life of decrease 19 min for D_2S_ versus 33 min for D_2L_)^46^. Although to a lower overall extent, Kim *et al*. found a similar 2: 1 ratio for D_2S_ (20%) versus D_2L_ (10%) receptor internalization. In this case, internalization was determined as the decrease in [³H]spiperone binding on the cell surface of transiently transfected CHO cells and found to be dependent on the expression of β-arrestin^24^. Using confocal microscopy, the same group observed a higher overall formation of endocytotic vesicles in D_2S_-expressing cells compared to the D_2L_-expressing counterparts upon agonist stimulation^24^.

Interestingly, our ELISA-based internalization studies in transiently transfected HEK cells did not reveal significant differences between D_2S_ and D_2L_ receptor internalization. Similar observations have been previously described by Thibault *et al*. who employed a nearly identical protocol. However, this group still found enhanced D_2S_ internalization compared to D_2L_ upon heterologous desensitization in the same cell line^34^. Together these results suggest crucial influences of the employed cell types, transfection conditions and probably receptor expression levels on the highly regulated internalization process.

Importantly, D_2S_ and D_2L_ receptors share an extremely high sequence homology differing in only 29 additional amino acids within the third intracellular loop (ICL3) of D_2L_^53^. Since the relatively long ICL3 has been previously reported to be important for D_2_ receptor desensitization and trafficking^4, 34, 54^, it is reasonable to assume that this region within ICL3 may attenuate receptor internalization and trafficking^5, 24^.

Previously, fluorescence microscopy based on immunohistochemistry staining has been used to measure the dynamics of agonist-induced D_2_ receptor internalization *in vitro* in intact cells^55^. Furthermore, receptor adaptations have been studied by means of simultaneous positron emission tomography (PET) and functional magnetic resonance imaging (fMRI), giving access to non-invasive methods of studying receptor internalization *in vivo* ^56^. The TIRF microscopy approach will allow to image native tissue with subcellular spatial resolution. Recent developments in the field of fluorescence microscopy will further promote the understanding of trafficking and regulation processes. As an example, multicolor imaging systems will facilitate a real time tracking of the dynamics of receptor-ligand complexes and their co-localization with various proteins involved in downstream signaling and internalization processes (e.g. agonist-receptor-G protein or agonist-receptor β-arrestin complexes) in living cells.

## Methods

### Cell culture

Chinese hamster ovary cells (CHO-K1) stably expressing human dopamine D_2L_^57^or D_2S_^57^ receptors were maintained in DMEM/F12 medium supplemented with 10% fetal bovine serum (FBS), 2 mM L-glutamine, 1% penicillin-streptomycin, and 800 µg mL^−1^ geneticin (all cell culture reagents purchased from Invitrogen/Thermo Fisher Scientific) at 37 °C, 5% CO_2_.

### Glass slide cleaning

The glass slide cleaning procedure was carried out as described previously^33^. In brief, 18 mm No. 1 glass slides (Assistent) were extensively cleaned to remove any background fluorescence. First, they were sonicated in a solution containing 12% Decon 90 for 1 h. After three washes with Milli-Q filtered water, they were further sonicated in a solution of 5 M NaOH for 1 h and washed again three times with Milli-Q filtered water. Glass slides were then dried followed by sonication in chloroform for 1 h. Cleaned glass slides were dried and stored in 100% ethanol until usage.

### Labeling and preparation for single-molecule TIRF microscopy imaging

24 h before the TIRF experiment, glass slides were placed in a 12-well plate and coated with 20 µg ml^−1^ fibronectin (Sigma-Aldrich) in sterile dPBS (without Ca^2+^/Mg^2+^), for 1 h at 37 °C. After coating, fibronectin was aspirated and the glass surface was rinsed once with sterile dPBS (without Ca^2+^/Mg^2+^). CHO cells stably expressing dopamine D_2L_ or D_2S_ receptors were seeded on coated glass slides in phenol red-free DMEM/F12 supplemented with 10% FBS and were allowed to adhere overnight at 37 °C, 5% CO_2_.

#### Fluorescent-ligand labeling

Cells were washed twice with phenol red-free DMEM/F12 supplemented with 10% FBS, labeled with a tenfold *K*_*i*_ value concentration of the corresponding fluorescent ligand and incubated unless otherwise stated at 37 °C and 5% CO_2_ for 1 h. Specific labeling of the fluorescent ligands was confirmed by preincubation with 10 µM spiperone (a potent non-fluorescent dopamine D_2_ receptor antagonist) for 2 h, followed by incubation with the fluorescent ligand as described above. Subsequently after labeling, cells were washed three times with phenol red-free DMEM/F12 supplemented with 10% FBS. Glass slides with labeled cells were placed in a custom-built imaging chamber (volume = 500 μL), washed twice with imaging buffer (137 mM NaCl, 5.4 mM KCl, 2.0 mM CaCl_2_, 1.0 mM MgCl_2_, and 10 mM HEPES, pH 7.4). Finally, the imaging chamber was refilled with fresh imaging buffer and mounted immediately on a microscope stage for TIRF microscopy imaging.

### TIRF microscopy

TIRF microscopy was carried out as described previously^33^. In brief, experiments were performed at room temperature (24.0 ± 0.3 °C) on a motorized Nikon TI-Eclipse inverted microscope equipped with a 100x, 1.49 NA oil-immersion objective. Fluorescent dyes were excited using a Nikon D-Eclipse C1 laser box with 561 nm laser for TIRF microscopy. The laser light was filtered by an excitation filter 561/14 nm and directed by a dichroic long-pass mirror (cut-off wavelength 561 nm). The emitted light was passed through an emission bandpass filter 609/54 nm (Semrock Rochester), and was projected onto a water-cooled (Polar Series Accel 250 LC, Thermo Scientific) EM-CCD camera at −98 °C (512 × 512 FT, DU-897, Andor, UK). The microscope control and image acquisition was performed by the NIS Elements software (Nikon Instruments). To ensure homogenous illumination, only the central quarter of the chip (300 × 300 pixel) was used for imaging analysis. The gain of the EM-CCD camera was kept constant at 300, binning at 1 × 1, BitDepth at 14 bits, readout speed 10 MHz. Image sequences (5–500 frames) were acquired with an exposure time of 50 ms, resulting in the frame rate of 19.32 fps (frames per second).

### Automated single particle tracking (ASPT)

An automated single particle tracking (ASPT) algorithm^58^ implemented in custom-written software, GMimPro (www.mashanov.uk), was used to identify and track individual fluorescent spots on sequential video frames to further determine their diffusive behavior. The procedure has been described previously in detail^33, 43, 59^.

### Calculation of the background corrected mean fluorescence intensity of single cells

Calculation of the mean background corrected fluorescence intensity *I(t)* (arbitrary units) at time *t* of single cells for internalization experiments were performed as described previously^33^. In brief, regions of interest (ROI) were drawn around the membrane of an individual fluorescent cell (ROI_cell_) and the background (ROI_background_) outside the cell using Fiji software^57^. The total mean intensity over the entire cell area (*I(t)*_*total cell*_) and the mean intensity over the background area (*I(t)*_*bg*_) were measured and *I(t)* were calculated as *I(t)* = *I (t)*_*total cell*_ − *I (t)*_*bg*_.

### Reductive treatment with sodium borohydride (NaBH_4_)

Samples of CHO cells stably expressing D_2L_ and D_2S_ receptors were prepared and labeled with the corresponding fluorescent ligands as described above. Labeled cells were treated with freshly prepared 30 mM NaBH_4_ solution for 5 min and washed once with dPBS (with Ca^2+^/Mg^2+^). Finally the imaging chamber was refilled with fresh imaging buffer and image acquisition was performed under TIRF illumination at 561 nm. The entire process was conducted on the microscope stage so that the same cells could be imaged before and after the reductive treatment.

### Internalization studies based on TIRF microscopy imaging

The mean fluorescence intensity (background corrected) of a fluorescent ligand labeled cell after NaBH_4_ treatment was used to estimate internalized fluorescence as a percentage of the mean fluorescence intensity (background corrected) of a labeled cell before NaBH_4_ reduction. Mean values and s.e.m were calculated from 9–24 cells from at least three independent experiments using Prism 6.0 (GraphPad Software, Inc.). Unpaired two-tailed Student’s *t*-tests were used to determine statistical significance.

### Measurement of emission spectra

To characterize the emission behaviour of the fluorescent ligand **1a** in the absence and presence of NaBH_4_ (30 mM, 5 min) emission spectra were recorded on a CLARIOstar multimode microplate reader (BMG Labtech, λ_ex_ of 530 nm) using black, clear bottom 96-well plates (Greiner Bio-One) and the ligand diluted in dPBS (with Ca^2+^/Mg^2+^, pH 7.4) to a concentration of 1 μM.

### Quantification of internalization by cell surface ELISA

Ligand-stimulated receptor internalization was quantified in analogy to a previously described procedure^34^. Briefly, HEK293T cells were transiently transfected in suspension with plasmids (pcDNA 3.1) encoding D_2S_R or D_2L_R fused to a FLAG-epitope at their N-terminus together with a plasmid encoding GRK2 (3:1 receptor to GRK2 ratio), using polyethyleneimine as transfection reagent^60^. Transfected cells were transferred into 48-well plates (65,000 cells/well) pretreated with Poly-D-Lysine and maintained at 37 °C, 5% CO_2_ for 48 h. After stimulation with the ligands dissolved in complete growth medium for 1 h at 37 °C, incubations were terminated by removal of the medium and fixation with 4% PFA (10 min, room temperature). Subsequently, cells were washed with washing buffer (150 mM NaCl, 25 mM Tris, pH 7.5), blocked for 1 h (3% skim milk powder in washing buffer) and incubated with the primary antibody (mouse anti-FLAG M2, 1:4,000, Sigma-Aldrich) for 1 h at room temperature. Cells were washed twice and blocked again before incubation with the horseradish peroxidase-conjugated secondary antibody for 1 h (HRP-rabbit anti mouse IgG, 1:20,000, Sigma-Aldrich). After three washing steps, 200 µL of a peroxidase substrate-containing solution (2.8 mM *o*-phenylenediamine in 35 mM citric acid, 66 mM Na_2_HPO_4_, pH 5.0) were added. Reactions were terminated after 30 min by addition of 200 µL 1 M H_2_SO_4_. From each well, 200 µL were transferred into a 96-well plate for absorbance readings at 492 nm on a CLARIOstar microplate reader. Data were analyzed by subtraction of the background (nontransfected cells) and normalization to control conditions (growth medium with 0.1% DMSO).

### β-Arrestin-2 recruitment assay

The measurement of β -arrestin-2 recruitment to activated-receptors was performed utilizing the PathHunter assay (DiscoverX, Birmingham, UK) as described previously^60^. HEK293 cells stably expressing the EA-tagged β-arrestin-2 fusion protein (provided by DiscoverX) were transiently transfected with the ProLink(ARMS2-PK2)-tagged dopamine D_2S_ or D_2L_ receptors, respectively, using Mirus TransIT-293 (Mobitech, Göttingen, Germany) transfection reagent. 24 h after transfection, cells were detached using Versene (Invitrogen) and 5,000 cells per well were seeded into white, clear bottom 384-well plates (Greiner Bio-One) and maintained at 37 °C, 5% CO_2_ for 24 h in assay medium. After incubation with different concentrations of test compounds (from 10^−12^ to 10^−5^ M final concentration) in duplicates for 5 h, the detection mix was added and incubation was continued for further 60 min. Chemiluminescence was determined on a CLARIOstar microplate reader. Resulting responses were normalized to the maximum effect obtained with quinpirole (100%) and the basal response (vehicle, 0%). Dose–response curves were calculated by nonlinear regression using the algorithms of Prism 6.0.

### Data availability

The data that support the findings of this study are available within the Supplementary Information files and/or from the corresponding authors upon request.

## Electronic supplementary material

Supplementary Information

Movie S1

Movie S2

Movie S3
