# Supplementary material for: Visualization of ligand-induced dopamine D2S and D2L receptor internalization by TIRF microscopy
Source: Sci Rep. 2017 Sep 7;7:10894. doi: 10.1038/s41598-017-11436-1 (PMC5589927; doi:10.1038/s41598-017-11436-1)
Supplement: Supplementary file 1 — Supplementary Information [file 41598_2017_11436_MOESM1_ESM.pdf]

Supplementary Information (SI)

Visualization of ligand-induced dopamine D_{2S} and D_{2L} receptor internalization by TIRF microscopy

Alina Tabor [1], Dorothee Möller [1], Harald Hübner [1], Johannes Kornhuber [2] and Peter Gmeiner [1,]*

[1] Department of Chemistry and Pharmacy, Emil Fischer Center, Friedrich-Alexander University Erlangen-Nürnberg, Schuhstraße 19, 91052 Erlangen, Germany

[2] Department of Psychiatry and Psychotherapy, Friedrich-Alexander University Erlangen-Nürnberg, Schwabachanlage 6, 91054 Erlangen, Germany

* Correspondence to: peter.gmeiner@fau.de

Supplementary Table

Supplementary Table S1. Receptor binding data and functional properties for quinpirole and the fluorescent ligands **1a**,**b** and **2a**,**b** at human D_{2L}, D_{2S} and D_3 receptors.

	**binding affinity**[a] [^{3}H]spiperone displacement			**β-arrestin-2 recruitment**[b] PathHunter Assay			
	**hD$_{2L}$**	**hD$_{2S}$**	**hD$_3$**	**hD$_{2L}$**		**hD$_{2S}$**	
**compound**	K_i [nM]	K_i [nM]	K_i [nM]	EC_{50} [nM]	E_{max} [%]	EC_{50} [nM]	E_{max} [%]
**quinpirole**	260 ± 40	70 ± 20	15 ± 4	110 ± 10	100 ± 1	79 ± 9	100 ± 1
**1a**	3.8 ± 2.8	7.8 ± 3.7	5.3 ± 2.3	n.d.	< 5	n.d.	< 6
**1b**	29 ± 9.6	42 ± 12	19 ± 4.3	n.d.	< 5	n.d.	< 6
**2a**	8.8 ± 6.1	7.1 ± 2.1	0.44 ± 0.20	400 ± 60	83 ± 7	240 ± 70	87 ± 4
**2b**	6.0 ± 2.7	7.7 ± 5.0	2.8 ± 0.5	230 ± 30	96 ± 4	230 ± 60	75 ± 7

[a]K_i values were taken from the authors' publications: Tabor et *al.* (2016, ref. 33) and Möller et *al.* (2014, ref. 60) and are given in nM ± standard deviation (s.d.) derived from n ≥ 2 individual experiments, each performed in triplicates. [b]β-arrestin-2 recruitment at D_{2L} and D_{2S} was normalized to the maximum response of the reference agonist quinpirole (100%) and vehicle (0%). Results are indicated as mean ± standard error (s.e.m.) of n ≥ 3 individual experiments, each performed in duplicates.

Supplementary Figures

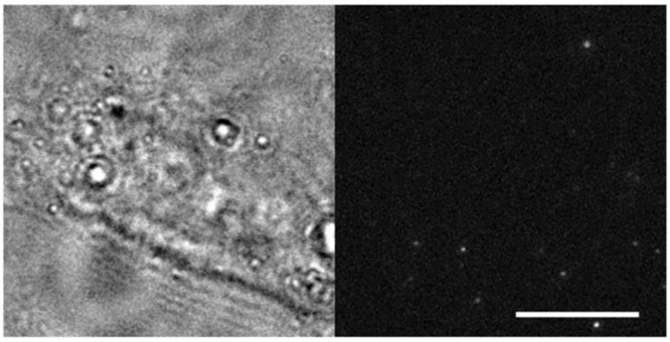

Supplementary Figure S1. Fluorescent ligand-induced D_{2S} receptor cluster formation is blocked by the pretreatment with the unlabeled antagonist spiperone. Representative brightfield (left) and TIRF microscopy image (right) of a CHO cell stably expressing D_{2S} receptors, pretreated with 10 µM spiperone for 2 h and subsequently incubated with the fluorescent agonist **2b** (10 nM) for 1 h. Neither cell-surface labeling nor fluorescent puncta were visible. Scale bar, 10 µm.

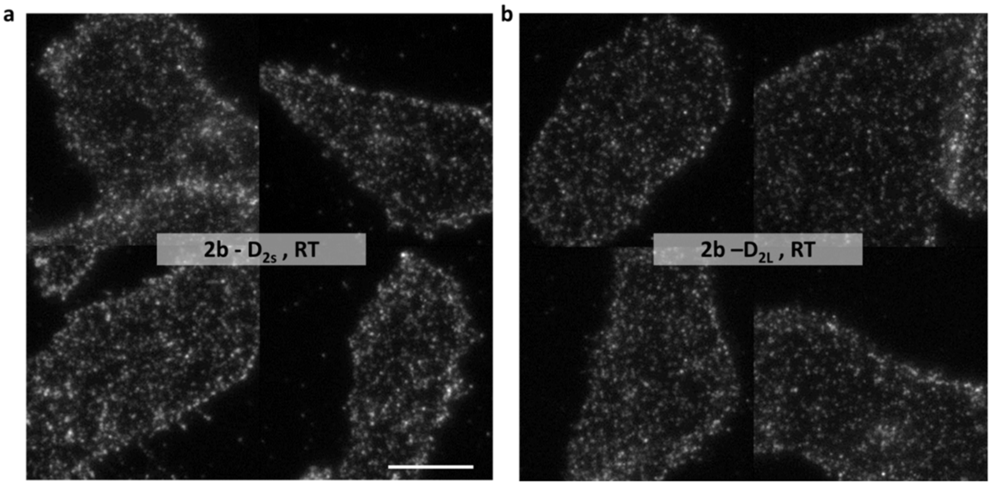

Supplementary Figure S2. Reduced formation of clustered fluorescent puncta of D_{2S} and D_{2L} receptors at ambient temperature. Representative TIRF microscopy images of CHO-cells stably expressing D_{2S} (**a**) or D_{2L} (**b**) receptors exposed to the fluorescent agonist **2b** (10 µM) for 1h at 22-24 °C. The number of intracellular fluorescent puncta is significantly reduced compared to the incubation temperature at 37°C (see Fig. 1d,e). Scale bar, 10 µm.

Supplementary Movies

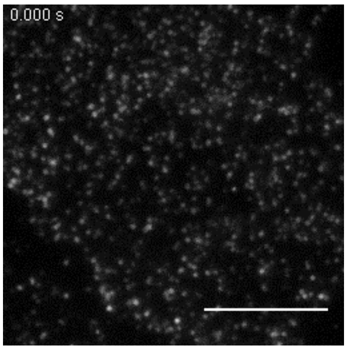

Supplementary Movie S1. First 100 frames of a TIRF microscopy recording of D_{2S} receptors labeled with the fluorescent antagonist **1b** in the plasma membrane of a single CHO cell (50 ms exposure time, frame rate of 19.32 fps). An image of this cell is shown in Figure 1b (main text).

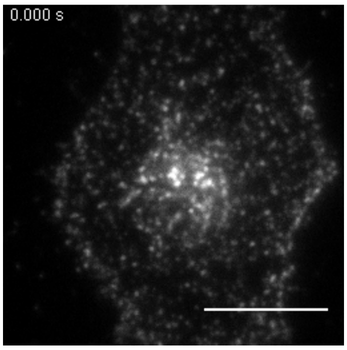

Supplementary Movie S2. First 100 frames of a TIRF microscopy recording of D_{2S} receptors labeled with the fluorescent agonist **2b** in the plasma membrane of a single CHO cell (50 ms exposure time, frame rate of 19.32 fps). An image of this cell is shown in Figure 1d (main text).

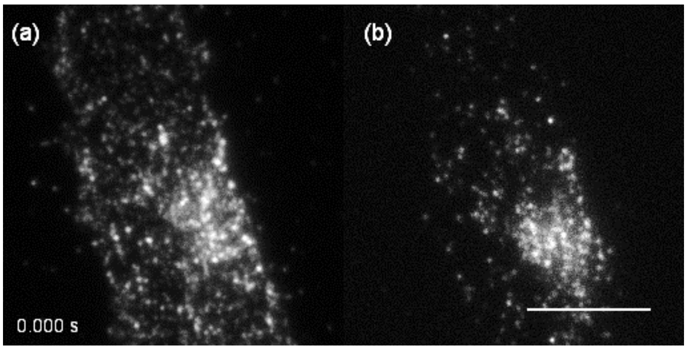

Supplementary Movie S3. First 30 frames of a TIRF microscopy recording of a single CHO cell stably expressing the D_{2S} receptor incubated with the fluorescent ligand **2b** for 1h before (**a**) and after treatment (**b**) with $NaBH_4$. (30 mM, 5 min). Scale bar, 10 µm. An image of this cell is shown in Figure 4a (main text).
